# Laparoscopic fundoplication for gastro-esophageal reflux disease: An 8 year experience

**DOI:** 10.4103/0972-9941.45205

**Published:** 2008

**Authors:** K P Balsara, C R Shah, M Hussain

**Affiliations:** Department of GI and Minimal Access Surgery, Bhatia, Breach Candy and Jaslok Hospitals, Mumbai, India

**Keywords:** Gastro esophageal reflux disease (GERD), laparoscopic fundoplication

## Abstract

**BACKGROUND:**

Laparoscopic fundoplication (LF) has become the operation of choice for patients who need surgery for gastro esophageal reflux disease (GERD). Several studies have shown that the long-term results with surgery for GERD are better than medical therapy. In this retrospective study, we outline our experience with LF over an 8 year period. We analyzed factors that would affect the results of surgery and help in a better selection of patients for the operation.

**MATERIALS AND METHODS:**

From 1999 to 2007, 107 patients underwent a LF. Eighty five patients had surgery for GERD and form the basis of this article. The other 22 patients had paraesophageal hernias and were excluded from the study. Pre-operative evaluation consisted of endoscopy, a barium study, esophageal manometry and 24h pH monitoring. Patients were followed up every 3rd month for the 1st year, twice in the 2nd year and then annually. Follow up was by personal interview or telephonic conversation. At the last follow up the results of surgery were graded as good or poor as per a scoring system. Those with a poor result were evaluated and re-operation advised when an anatomical problem caused the poor result. Subjective, objective and technical variables were analyzed which could affect the outcome of surgery.

**RESULTS:**

In 84 patients, the operation was completed by laparoscopic access. One patient with bleeding was converted to open surgery. There were 5 intra-operative complications; 3 pnemothoracis, 1 esophageal perforation and 1 gastric fundus perforation. There was no mortality. Two patients underwent re-operation, 1 for delayed gastric emptying and 1 for dysphagia. Seventy four patients have been followed up from 7 months to 8 years. Eleven have been lost to follow up. Fifty seven patients (77%) have had a good result from surgery. Seventeen (23%) had a poor result; of these there were 4 wrap failures, 1 delayed gastric emptying and 1 excessive gas bloat as the cause. In 11 patients, there was no apparent cause of a poor result. Individual variables which predicted a good response to surgery (*P*<0.5); were a good response to proton pump inhibitors (PPis), volume reflux and a pH score of more than 14.

**CONCLUSION:**

LF gives good long-term relief of symptoms in patients with GERD. Strict selection criteria are necessary to optimize the results of surgery. Poor selection will result in a patient who is no better, or often worse than before surgery.

## INTRODUCTION

Gastro esophageal reflux disease (GERD) is an uncommon problem in India as compared to the west.[1,2] Patients are always initially managed by proton pump inhibitors (PPis), prokinetic drugs and life style changes. Surgery is indicated when these measures fail, when complications develop or when patients’ opt for surgical treatment rather than lifelong medication. Several studies have shown that operative intervention offers a better quality of life as compared to medical treatment.[3,4] With the advent of minimal access surgery, the popularity of anti-reflux surgery has grown enormously.[5] Though most authors have reported good long term results,[6,7] some have questioned the efficacy and durability of this procedure.[8]

In this paper, we outline our experience of laparoscopic fundoplication (LF) for GERD over an 8 year period along with a medium to long term follow up.

## MATERIALS AND METHODS

From 1999 October to October 2007, 107 patients underwent LF. Eighty five patients had GERD with or without a type 1 hiatal hernia were retrospectively analyzed. The other 22 patients had mixed (type 3) hiatal hernias, with predominant chest symptoms and were excluded from this study. There were 58 males and 27 females, with age range from 7 to 79 years (mean 56 years). All patients had been on PPis for periods ranging from 3 to 9 years. The response to PPis was graded as good, poor or indeterminate. The symptoms on presentation were: heartburn in all, regurgitation in 43 and volume reflux in 39 patients. Volume reflux was considered separately when patients regurgitated large quantities of gastric acid and ingested food. Dysphagia, respiratory symptoms, and dental problems occurred in 8, 5, and 1 patients, respectively. Pre-operatively all patients were evaluated by endoscopy and a barium study. The esophagitis was graded by the Savary Miller classification.[9] Barrett's metaplasia was noted in addition. Forty-eight patients underwent esophageal manometry and 46 patients had 24h pH monitoring done. This has now become our standard pre-operative protocol. Manometry was done using a 16 channel high-resolution catheter; lower esophageal sphincter (LES) pressure (*n*:10-20 mmHg) and motility (<50% forward propulsive waves on wet swallows was poor motility) were measured. Twenty four hour ambulatory pH monitoring was done using an antimony electrode (Sandhill scientific. USA). The data was recorded on a digitrapper and then analyzed. A composite Demeester score of more than 14 was considered significant for abnormal reflux. Individual scores of pH were not used in calculations.

The indications for surgery were: a) recurrent reflux while on PPis, b) volume reflux, C) recurrent esophageal strictures, and 4) patients wish to stop PPis.

Patients with strictures were dilated to a minimum of 32FG before surgery.

### Operative procedure

Briefly; the procedure was divided into the following steps a) Crural identification, b) esophageal mobilization to bring down 3 to 4 centimeters of esophagus into the abdomen, c) complete division of all short gastric vessels, d) crural repair using 1/0 prolene, e) 360 degree, short floppy fundal plication using 2/0 ethibond suture (ethicon surgical) or a 270 degree Toupet procedure. A harmonic shear (ethicon endosurgery) was used for most of the dissection. The hepatic branch of the anterior vagus was preserved when easily feasible. An esophageal bougie of 50FG (Pilling corp. USA) was used before the plication in 35 cases. We have abandoned routine use in all patients. Oral liquids were started the day after surgery and a soft mashed diet from the 2^nd^day. This was continued for 3 weeks post surgery after which the patient could consume a normal diet. Patients were asked to use a straw to drink liquids to reduce aerophagy for 3 months after surgery.

Postoperative follow up was every 3^rd^ month for the 1^st^ year, twice a year for the second year and these were by personal interview only. Subsequent annual follow up was either by telephonic conversation or personal interview. At the last follow up before compilation of data, a fixed set of questions were asked and each answer was assigned a score [Table 1]. A higher ordinal score representing a better result. We have used a simple scoring system. Though not validated, the questions asked are relevant and patients find it easy to answer.

**Table 1:** Questionnaire used for follow up

Questions			
Relief of symptoms	0	50%	80% +
	(0)	(1)	(2)
PPi use	regular	occasional	nil
	(0)	(1)	(2)
Side effects			
(Gas bloat, dysphagia, flatulence)			
	severe	occasional	Nil
	(0)	(1)	(2)
Would recommend the procedure	no	maybe	yes
	(0)	(1)	(2)

Figures in parenthesis represent scores, Total score of 6 and above was a good result, Total score <6 was a poor result

The follow up period ranged from 7 months to 8 years (mean 4.6 years).

Those with a poor result were evaluated with an endoscopy and a barium study. If necessary, esophageal manometry and 24h pH monitoring were added. Patients who had anatomical wrap failures were advised re-operation.

### Statistical methods

Binary logistic regression was used to evaluate independent subjective, objective and technical variables on the outcome of surgery which was the dependant variable. Chi squared test was used to calculate the *P* value, which was significant if less than 0.05 [Table 2].

**Table 2:** Analysis of variables and their effect on outcome

Variable	Nos.	Response to surgery		*P*	OR	CI
						
		Poor	good			
PPi response						
Good	43	5	38			
Poor/ inderm.	31	12	19	0.009	0.208	0.064-0678
Vol. reflux						
+ve	39	4	35			
-ve	35	13	22	0.009	5.17,	1.45-17.88
Endosc. grade						
1-2	53	13	40			
3-4	21	4	17	0.61	1.38,	0.39-4.58
Hiatus hernia						
Nil	20	7	13			
Up to 2 cms	37	6	31			
2 to 5 cms	17	4	13	0.36	1-43,	0.65-3.13
pH scores						
<14	6	5	1			
>14	40	7	33	0.007	23.5,	2.37- 234.
LES press						
Low <10mmHg	30	8	22			
N: 10-20	18	3	15	0.42	1.18,	0.41-7.99
Motility						
Poor	16	6	10			
Normal	32	11	22	0.83	1.14,	0.34-3-98
Bougie used						
Yes	30	7	23			
No	44	10	34	0.95	1-035,	0.34-3.18

## RESULTS

Forty three patients had a good response to PPis while 31 had a poor or inderminate response.

Pre-operative evaluation revealed a hiatus hernia in 63 patients, ranging in size from 1 to 5 centimeters. Esophagitis was of grade 1 in 15 patients, grade 2 in 48, grade 3 in 17 and grade 4 in 5. Five had Barrett's metaplasia but none showed dysplastic changes. Manometry in 48 and pH monitoring in 46 patients were done. Thirty patients had a low LES pressure (normal 10-20 mmHg) and 16 had dysmotility. Two patients had severe dysmotility (<3 propagated waves on wet swallows). Neither of these patients had achalasia and one of them was also evaluated for scleroderma, which was negative. Forty patients had a composite pH score of more than 14.

Five patients with strictures were dilated to a minimum of 32FG before surgery.

In 84 patients the operation was completed by laparoscopic technique, 1 was converted to a laparotomy due to short gastric vessel bleeding. One patient had a 270 degree Toupet procedure; all the others had a 360 Nissen's fundoplication. There were 5 intra-operative complications. Three pnemothoracis which needed no drains, 1 gastric fundal perforation which was sutured laparoscopically and 1 esophageal perforation caused while passing an esophageal bougie. This patient presented with an empyema 6 days after surgery which needed drainage; no perforation could be demonstrated on oral contrast study. There were no deaths in this series.

Two patients had re-operation; 1 for poor gastric emptying probably due to a primary gastroparesis; she had a gastrojejunostomy. The other patient had persistent dysphagia and was found to have severe dysmotility on manometry. Her preoperative manometry had also shown a severe dysmotility. She had the wrap revised from 360 to 180 degrees, 2 months after the initial operation.

A total of 74 patients were contactable at the end of the study period, eleven were lost to follow up. A majority of those lost to follow up were from the earlier years of surgery. Fifty seven patients (77%) reported a good result as per our scoring system. Seventeen had a poor result. All 17 were re-evaluated by endoscopy and barium study; 4 had esophageal physiology tests in addition. In three patients, the stomach had migrated into the chest and all 3 had recurrent esophagitis and abnormal pH scores. They refused re-operation and are on PPis. Of the 2 patients who had revision surgery; the one with the wrap revision reported a 50% benefit in dysphagia 5 months after the correction: while the other patient felt she had no benefit at all. One patient had recurrent severe gas bloat which led to a poor outcome. The other 11 patients with a poor result had normal endoscopy and barium studies.

On analyzing these 11 patients, we found that all had an indeterminate response to pre-operative PPi use. The esophagitis was of grade 1 in 9 and of grade 2 in the other 2 patients. Barium studies showed no reflux in any, though 5 had small hiatal hernias between 1 and 2 centimeters. None of these patients had any physiologic tests as they were not available to us at the time.

Of the variables used to predict a good surgical outcome, only a good response to pre-operative PPis, volume reflux and a pH score of more than 14 were significant, *P* value of less than 0.05 [Table 2].

## DISCUSSION

With the advent of laparoscopic surgery, the rate of LF rose sharply in the US and then declined from 1999 to 2003.[5] In the UK the referrals for surgery for GERD seem to be growing steadily over the years.[10] No similar data are available in India, but our experience suggests that referral patterns have remained static over the last 8 years.

With easy availability of esophageal physiology, we have been able to do a more comprehensive pre-operative evaluation on more than 50% of our patients. This has allowed us to become more stringent in our patient selection. Manometry is necessary to rule out an achalasia and identify an underlying motility disorder.

We have not tailored the fundoplication depending on esophageal motility and this practice has largely been abandoned by most authors.[11] Twenty four hour pH monitoring of the esophagus is an ideal way of quantifying reflux. Though not mandatory for patients with erosive esophagitis, it serves as a good baseline study.

Our technique has changed marginally since inception. We have switched to using 1/0 polypropylene suture for crural repair from 2/0 Ethibond; 1/0 suture is stronger than 2/0 but unfortunately not available in Ethibond material in India. We have stopped using an esophageal bougie before the wrap, but rather rely on complete fundal mobilization and visual impression of the looseness of the wrap.

Defining outcomes after antireflux surgery have lacked uniformity.[17] Authors have used varying methods of defining outcomes; from symptomatic benefit, continued PPi use to more elaborate quality of life scores, endoscopy and esophageal physiology. With this background, we devised a simple easy to use scoring system, not previously validated, for this study. Depending on the result, we further evaluated these patients.

Seventy four patients were available for follow up at the end of the study period (90%). Fifty seven had a good outcome and 17 had a poor result. These results are inferior to those reported by others.[12,13] Of the 17 patients with a poor result, 3 had anatomical failures in the form of intrathoracic wrap migration. One patient with gastroparesis had presented with vomiting. After surgery, her symptoms worsened and she needed a gastro-jejunostomy. Gastric dysmotility is often difficult to identify; a patient who has vomiting instead of regurgitation needs to be carefully evaluated before surgery. Radionuclide gastric emptying can help in identifying this problem. Gas bloat after surgery is a common side effect and occurred in nearly 30% of our patients. In one patient, it was severe and led to a poor overall outcome. We advise our patients to use a straw to drink liquids after surgery for 3 months. This seems to reduce aerophagy and gas bloat. Persistent dysphagia occurred in one patient who had low LES pressures and poor esophageal peristalsis on manometry pre-operatively. Scleroderma was ruled out by appropriate biochemical tests. She underwent surgery again and the wrap was converted from a 360 to a 180 posterior wrap. After redo surgery her dysphagia resolved by 50%, but not completely and has remained as such 5 months after the correction.

No other patient complained of dysphagia and this low incidence is in accordance with other studies.[10,11,14] Eleven patients with a poor result had no abnormality on endoscopy or a barium study. Not all these patients could define their response to PPi use before surgery. None had erosive esophagitis and 5 had small hiatal hernias. Clearly, there was a need for esophageal physiology to get a more objective evaluation of the problem. Unfortunately, these tests were not available to us at the time. These patients continue to follow up with us, their symptoms persist and some of them have the added burden of mild gas bloat. We feel that these failures were indeed due to inappropriate patient selection.

Several authors have emphasized the need for complete evaluation of patients before surgery.[14–16] Obesity, underlying psychological problems and atypical symptoms may all be indicators of a poor outcome. A poor symptom response to PPis and a normal pH score should also guard the surgeon from rushing in to do an anti-reflux operation. Our study showed that indicators to a good surgical outcome are; a good response to pre-operative PPis, volume reflux, and a pH score of more than 14.

## CONCLUSION

Laparoscopic fundoplication for gastro esophageal reflux disease is an effective and durable operation with a good long-term outcome. We remain enthusiastic about this operation but emphasize on a stringent pre-operative evaluation. Poorly selection can render a patient with no symptom relief and often with considerable side effects.
